# Antioxidant, antibacterial, and molecular docking of methyl ferulate and oleic acid produced by *Aspergillus pseudodeflectus* AUMC 15761 utilizing wheat bran

**DOI:** 10.1038/s41598-024-52045-z

**Published:** 2024-02-07

**Authors:** Ahmed Mohamed Ahmed Ali Ramadan, Sabry Ahmed Hussein Zidan, Reda Mohamed Shehata, Hussein Hosny EL-Sheikh, Fuad Ameen, Steven L. Stephenson, Osama Abdel-Hafeez Mohamed Al-Bedak

**Affiliations:** 1 Department of Botany & Microbiology, Faculty of Science, Al Azhar University, Cairo, Egypt; 2 Department of Pharmacognosy, Faculty of Pharmacy, Al-Azhar University, Assiut Branch, Assiut, 71524 Egypt; 3 The Regional Center for Mycology and Biotechnology (RCMB), Al Azhar University, Cairo, Egypt; 4 Department of Botany & Microbiology, College of Science, King Saud University, 11451 Riyadh, Saudi Arabia; 5 Department of Biological Sciences, University of Arkansas, Fayetteville, USA; 6 Assiut University Mycological Centre, Assiut University, Assiut, 71511 Egypt

**Keywords:** Biotechnology, Microbiology

## Abstract

Secondary metabolites (SMs) are the primary source of therapeutics and lead chemicals in medicine. They have been especially important in the creation of effective cures for conditions such as cancer, malaria, bacterial and fungal infections, neurological and cardiovascular problems, and autoimmune illnesses. In the present study, *Aspergillus pseudodeflectus* AUMC 15761 was demonstrated to use wheat bran in solid state fermentation (SSF) at optimum conditions (pH 7.0 at 30 °C after 10 days of incubation and using sodium nitrate as a nitrogen source) to produce methyl ferulate and oleic acid with significant antioxidant and antibacterial properties. Gas chromatography-mass spectrometry (GC–MS) analysis of the crude methanol extract revealed eleven peaks that indicated the most common chemical components. Purification of methyl ferulate and oleic acid was carried out by column chromatography, and both compounds were identified by in-depth spectroscopic analysis, including 1D and 2D NMR and HR-ESI–MS. DPPH activity increased as the sample concentration increased. IC_50_ values of both compounds obtained were 73.213 ± 11.20 and 104.178 ± 9.53 µM, respectively. Also, the MIC value for methyl ferulate against *Bacillus subtilis* and *Staphylococcus aureus* was 0.31 mg/mL, while the corresponding MIC values for oleic acid were 1.25 mg/mL and 0.62 mg/mL for both bacterial strains, respectively. Molecular modeling calculations were carried out to reveal the binding mode of methyl ferulate and oleic acid within the binding site of the crucial proteins of *Staphylococcus aureus*. The docking results were found to be well correlated with the experimental data.

## Introduction

Currently, infectious diseases related to bacteria, fungi, and viruses represent a significant issue for global health^1^. Worldwide, acute respiratory infections (ARIs), intestinal infections, HIV/AIDS, tuberculosis, and malaria annually account for roughly 4, 3, 1.8, 0.7, and 1.3 million deaths, respectively^2,3^. Rising microbial resistance to conventional medication has had an significant impact on research into novel substances that demonstrate broad-spectrum antibacterial activity^4^.

Secondary metabolites are synthesized by a range of plants, animals, and microorganisms. Fungi, which are particularly prolific sources of bioactive secondary metabolites^5^**,** continue to be among the most important organisms investigated for therapeutic agents and lead compounds in medicine. They have been especially important in the development of effective therapies for cancer, malaria, bacterial and fungal infections, neurological and cardiovascular diseases, and autoimmune disorders^6^**.**

Fungi create a broad array of secondary metabolites, some of which are harmful to humans, plants, animals, and the environment^7^. In contrast, they also serve as a reliable source for the creation of a number of beneficial products, including enzymes^8–10^, biodiesel^11^, fatty acids^12^, and mycotoxins^13^, as well as being used to make pharmaceutical, agrochemical, and cosmetic commodities. Despite the fact that many secondary metabolites generated from fungi have been discovered previously, there are still many others that have yet to be discovered. Only a few secondary metabolites have been recognized from fungi, although there have been more than species of 150,000 fungi identified thus far (Species Fungorum database). This is because it is difficult to find and recognize new secondary metabolites^7^.

Methyl ferulate, which was first detected from the medicinal plant *Stemona tuberosa*, has the ability to cross cell membranes and enter the brain; it also exhibits anti-free radical characteristics^14,15^. Methyl ferulate has also been found in a diverse range of fruits such as oranges and tomatoes, and in some cereals such as rice and corn^16–19^. Because of its low toxicity and diminishing oxidation activity, it has been utilized effectively as a food additive^20,21^ in cosmetics, as well as in health care and skin care products^14^. Oleic acid is a natural product that has recently become commonly used to prevent food from oxidizing and it also has great antimicrobial potential value against many fungi and bacteria^22,23^. Oleic acid, which makes up around 80% of the total fatty acids in virgin olive oil^24^, is used to decrease cholesterol and reduce inflammation in order to avoid heart diseases^25–27^.

Globally, there is a huge accumulation of agricultural and industrial waste. Nevertheless, because these wastes usually include a substantial content of carbohydrates, minerals, and proteins along with cellulose (30–40%), hemicellulose (20–40%), and lignin (20–30%)^28^, they should not be regarded as "wastes" but rather as "raw materials" for other industrial processes^29^. Notwithstanding the fact that they are currently underutilized in Egypt, a variety of agro-industrial wastes have been employed as substrates in the solid state fermentation process due to their availability, low cost, environmental friendliness, prolonged shelf life, and simplicity of downstream processing^30,31^. As a result, the present study focused on the production of methyl ferulate and oleic acid by *Aspergillus pseudodeflectus* AUMC 15761 from wheat bran under solid state fermentation conditions as well as on methods to purify, identify, and employ these metabolites as antioxidant and antibacterial agents. In addition, the binding mode of the two obtained compounds within the binding site of the crucial proteins of *Staphylococcus aureus* was investigated using molecular modeling computations.

## Results

### Morphological and molecular identification of *Aspergillus pseudodeflectus* strain

**Figure 1:**
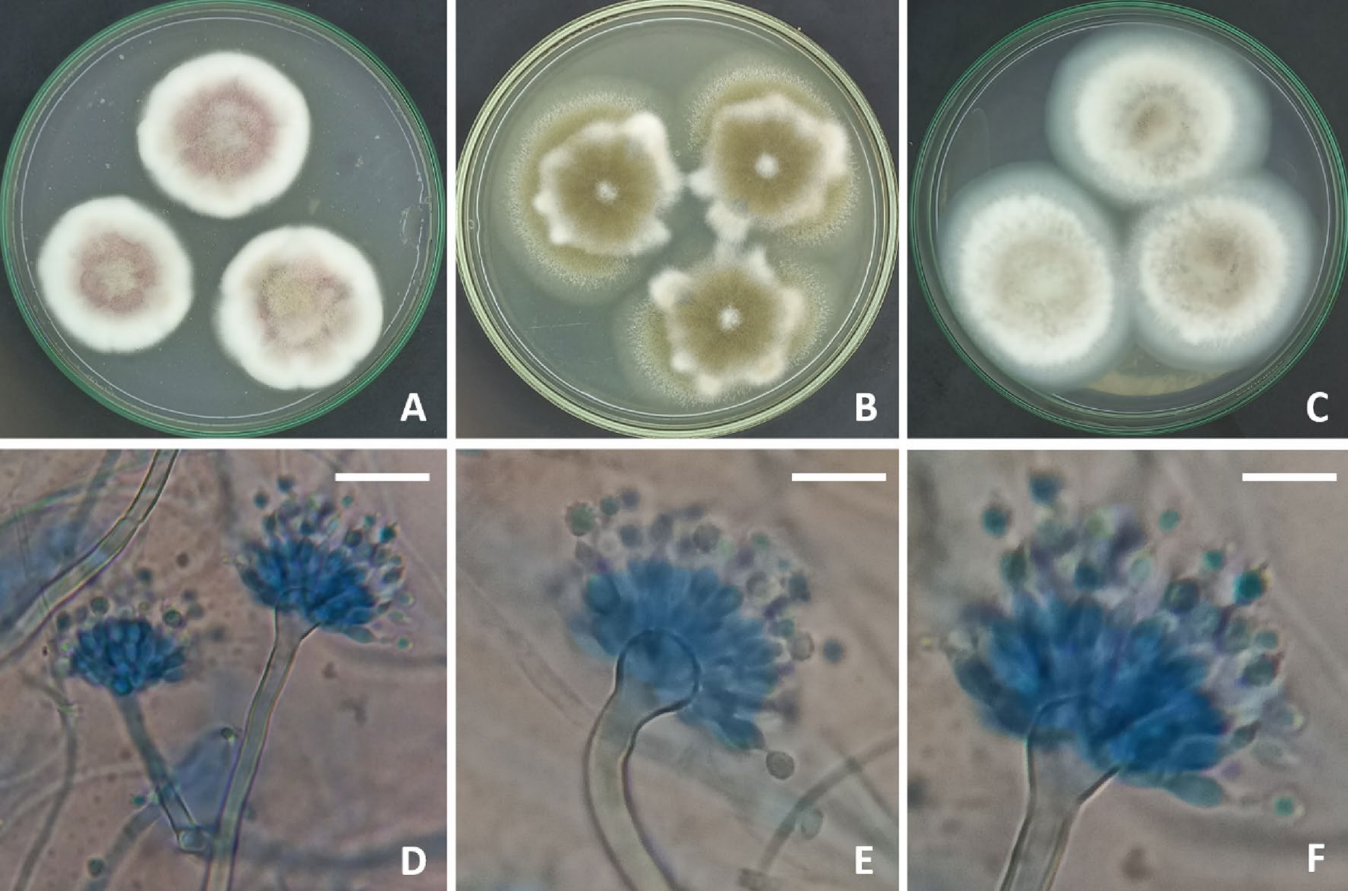
*Aspergillus pseudodeflectus* AUMC 15761 (**A**–**C**), 7—day-old colonies on Cz, MEA, and CYA at 25 °C. (**D**–**F**), Conidiophores and biseriate, columnar conidial heads. (Scale bars = 10 µm).

The morphological characteristics of the *Aspergillus* strain used in this study shared the identical features of *A. pseudodeflectus* as having radiate, brown conidial heads. Stipes 35–200 × 2.5–3.5 µm. Vesicles globose to clavate, 4–12 µm. Conidia globose to ellipsoidal, brown, 3.5–5 µm. Hülle cells absent (Fig. 1).

**Figure 2:**
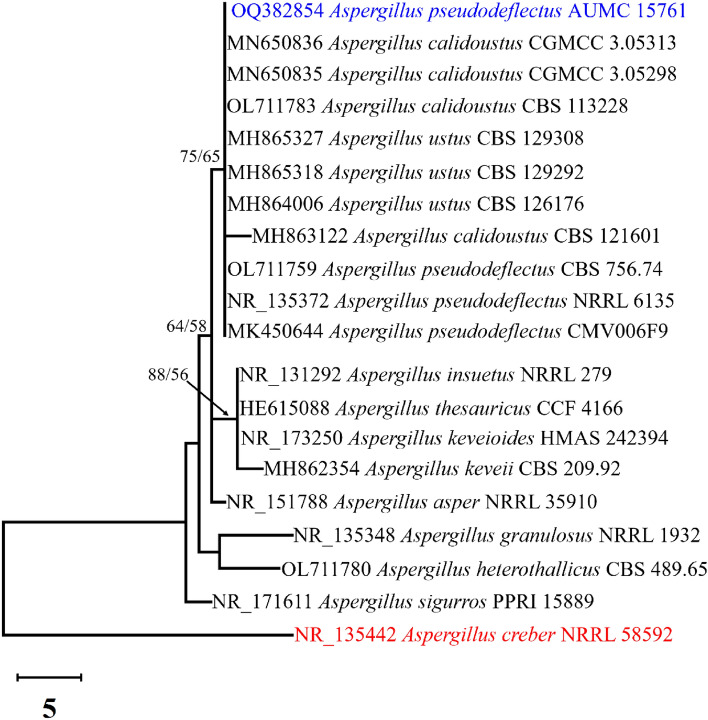
The most parsimonious phylogenetic tree obtained from a heuristic search (1000 replications) of the ITS sequence of *A. pseudodeflectus* AUMC 15761 (in blue) compared to other closely similar ITS sequences belonging to genus *Aspergillus*: section Usti in GenBank. Bootstrap support values for ML/MP ≥ 50% are indicated near the respective nodes. The tree is rooted to *Aspergillus creber* BRRL 5892 as an outgroup (in red).

Phylogenetic analysis based on ITS sequencing was employed to confirm the identification of the strain. The final ITS data set contained 20 sequences that produced 616 characters, of which 505 characters could be correctly aligned, 46 characters were counted as variable, and 8 as informative. The Tamura 3-parameter using a discrete Gamma distribution (T92 + G) was the perfect model used to represent the relationship among taxa. The maximum Parsimony method yielded 10 trees, the most parsimonious of which (Fig. 2) has a tree length of 61, the highest log likelihood of − 1180.74, consistency index of 0.733333, retention index of 0.818182, and a composite index of 0.600000 is shown in Fig. 2.

### Production of secondary metabolites by *Aspergillus pseudodeflectus* AUMC 15761 utilizing lignocellulosic wastes

**Figure 3:**
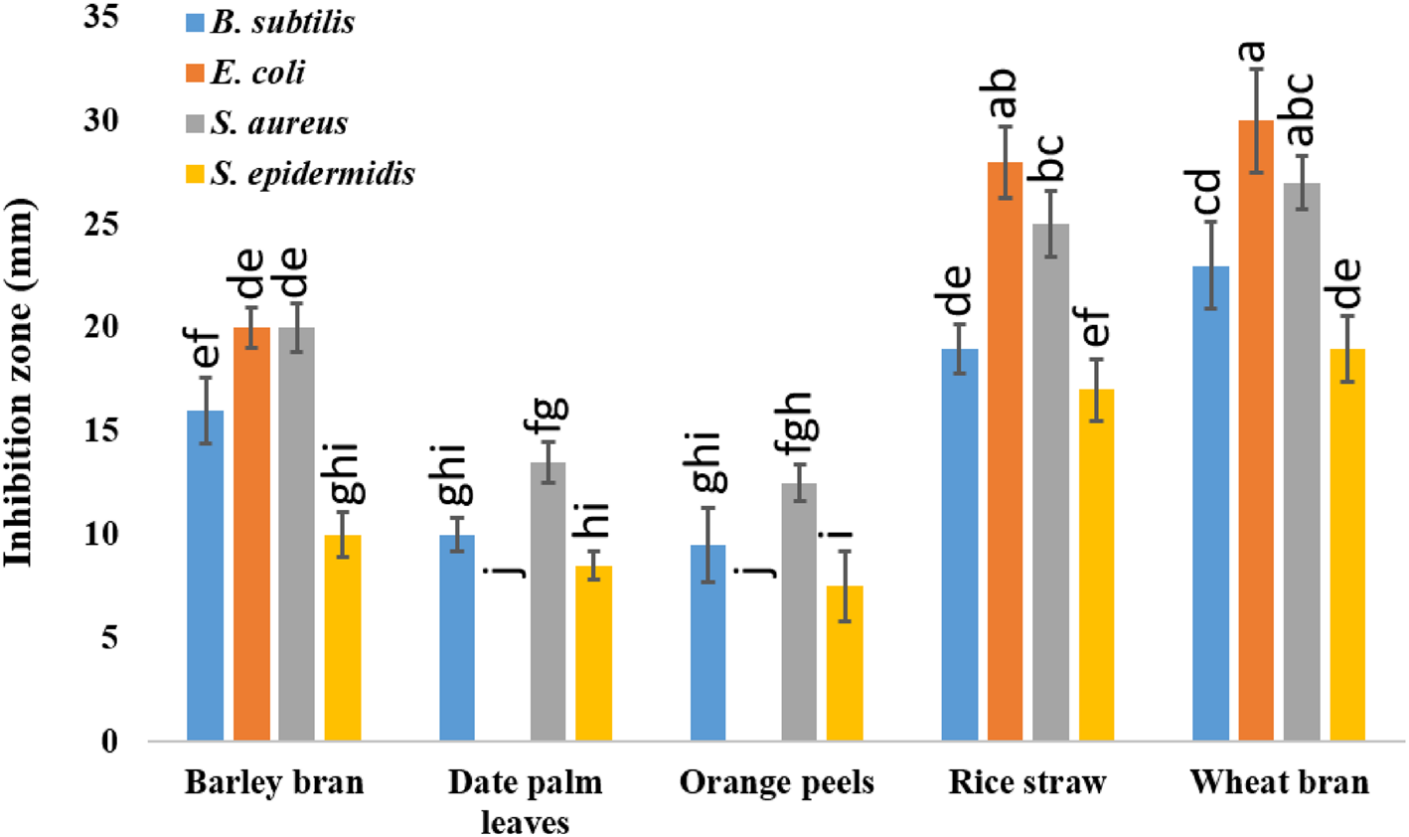
Antibacterial activity (as inhibition zone), of the crude extract of different lignocellulosic wastes fermented by *A. pseudodeflectus* AUMC 15761 under SSF. Mean values (± SD) on the bars of the graph with different letters are significantly different (*p* ≤ 0.05; *n* = 3).

The five lignocellulosic wastes (barley bran, date palm leaves, orange peels, rice straw, and wheat bran) used in SSF were fermented in different proportions by *A. pseudodeflectus* AUMC 15761. Wheat bran produced the most powerful crude extract that exhibited the largest inhibition zone against the examined strains. Following *Escherichia. coli* in terms of severity of impact were *Bacillus subtilis*, *Staphylococcus aureus*, and *Staphylococcus epidermidis* (Fig. 3).

### GC–MS analysis

**Figure 4:**
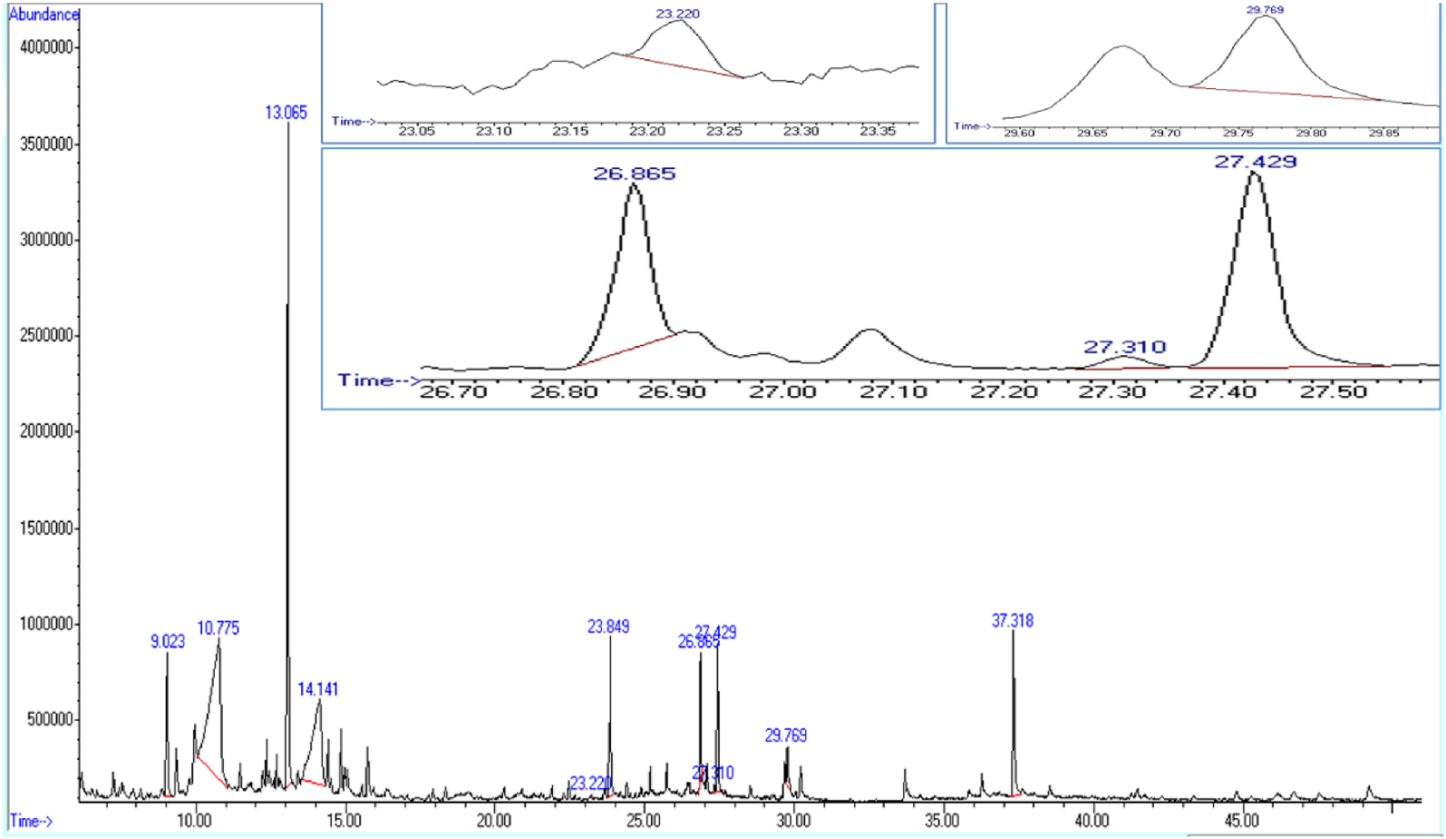
GC–MS chromatogram of the methanol extract wheat bran fermented by *A. pseudodeflectus* AUMC 15761 under SSF.

The GC–MS analysis of the methanol extract was carried out to evaluate its potential components since wheat bran extract was determined to be the most promising. Based on the retention time, molecular weight, and fragmentation pattern of the most prominent chemicals, the current results revealed eleven peaks. These were shown to have retention times of 23.222, 27.429, and 29.769 min, respectively, for *trans*-Ferulic acid, 3-(2, 5-Dimethoxyphenyl) propionic acid, and oleic acid (Table 1; Fig. 4).

**Table 1:** GC–MS spectral analysis of the chemical compounds detected in the methanol extract of wheat bran fermented by *A. pseudodeflectus* AUMC 15761 under SSF.

Retention time (min)	Name of the compound	Molecular weight	Molecular formula
37.318	1,8-dihydroxy-3-methoxy-6-methylAnthraquinone	270.28	C_16_H_14_O_4_
9.023	2-Hydroxycyclopent-2-en-1-one	98.1	C_5_H_6_O_2_
27.429	3-(2,5-Dimethoxyphenyl)propionic acid	210.23	C_11_H_14_O_4_
27.310	3-(3',5'-Dimethoxy-4'-hydroxyphenyl)-E2-propenal	212.24	C_11_H_16_O_4_
23.849	4-((1E)-3-Hydroxy-1-propenyl)-2- methoxyphenol	180.2	C_10_H_12_O_3_
13.065	4-Vinylphenol	120.15	C_8_H_8_O
14.141	Erythritol	122.12	C_4_H_10_O_4_
23.220	*trans*- Ferulic acid	194.18	C_10_H_10_O_4_
10.775	Glycerin	92.09	C_3_H_8_O_3_
26.865	Hexadecanoic acid	256.42	C_16_H_32_O_2_
29.769	Oleic acid	282.5	C_18_H_34_O_2_

### Optimization of production conditions of the bio-active secondary metabolites using wheat bran

**Figure 5:**
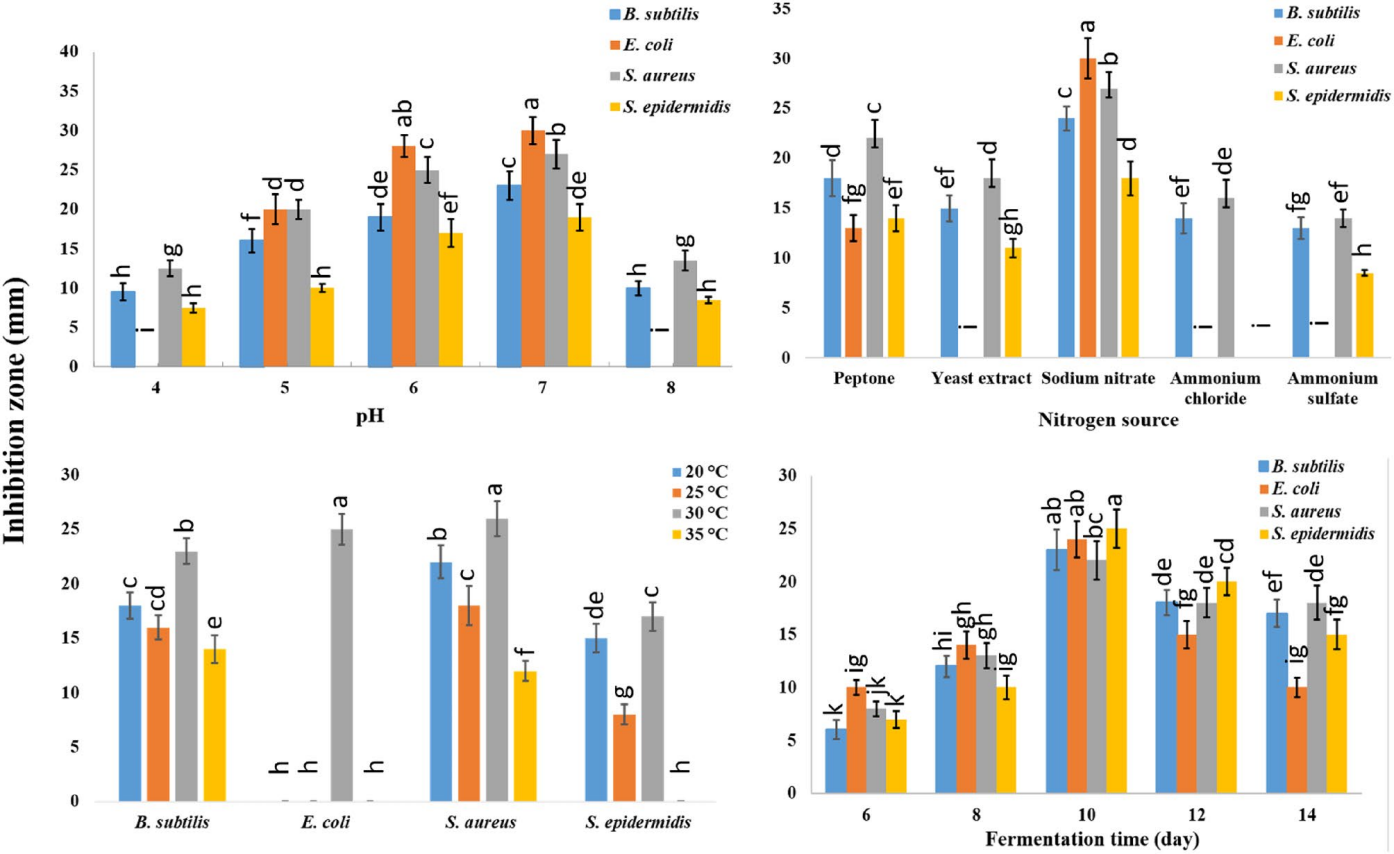
Optimization of fermentation conditions of the bio-active secondary metabolites using wheat bran fermented by *A. pseudodeflectus* AUMC 15761 under SSF*.* Mean values (± SD) for bars on the graph with different letters are significantly different (*p* ≤ 0.05; *n* = 3).

Based on a one factor at a time (OFAT) analysis, the results obtained revealed that *A. pseudodeflectus* AUMC 15761 could produce the most bio-active secondary metabolites with the greatest effect against the tested bacteria at pH 7.0 using sodium nitrate as a nitrogen supply after 10 days of incubation at 30 °C. These optimal conditions were found to cause the greatest inhibition of the four tested bacterial strains. The inhibition zones were 20.5 ± 1.3, 37.9 ± 1.7, 27.0 ± 1.8, and 19.0 ± 1.7 mm for *B. subtilis*, *E. coli*, *S. aureus*, and *S. epidermidis*, respectively (Fig. 5).

### Production and purification of bio-active secondary metabolites by column chromatography

*Aspergillus pseudodeflectus* AUMC 15761 could ferment 500 g of wheat bran in SSF, and 90.0 g (18%) of crude extract were produced. Using *n*-hexane, dichloromethane (DCM), and 0–100% gradients of MeOH in the DCM solvent system, twelve fractions (F1–F12) were obtained after fractionation by means of the VLC column (Table 2), Fraction F3 (750 mg), which was eluted by DCM: MeOH (95: 5) had the highest inhibition (23.0 ± 1.8, 32.9 ± 1.6, 42.5 ± 1.4, and 39.4 ± 1.5 mm) against *B. subtilis*, *E. coli*, *S. aureus*, and *S. epidermidis*, respectively. Consequently, it was subjected to purification by silica gel open column (1 × 100 cm). Nine sub-fractions (F3S1–F3S9) were subsequently produced as a result, with F3S8 (40 mg) and F3S4 (100 mg) being the two most active. Further purification using a 0.5 × 25 cm open column of both fractions, produced a pure compound **2** (40 mg) from F3S4, while F3S8 required a final purification step by preparative TLC plates (60 PF_254_) to obtain the pure compounds **1** (8.0 mg).

**Table 2:** The antibacterial potential of the fractions obtained by VLC column of wheat bran fermented by *A. pseudodeflectus* AUMC 15761 under SSF.

*Fractions*	Tested microbes			
	*B. subtilis*	*E. coli*	*S. aureus*	*S. epidermidis*
F1- *n*-hexane	17.0 ± 1.8^fg^	0^i^	0^i^	0^i^
F2- DCM	0^i^	0^i^	17.5 ± 1.5^fg^	0^i^
F3	23.0 ± 1.8^e^	32.9 ± 1.6^c^	42.5 ± 1.4^a^	39.4 ± 1.5^b^
F4	0^i^	0^i^	0^i^	0^i^
F5	16.5 ± 1.2^gh^	31.5 ± 1.7^d^	30.5 ± 1.5^d^	33.4 ± 1.6^c^
F6	18.3 ± 1.3^f^	15.7 ± 0.9^h^	17.5 ± 1.2^fg^	0^i^
F7	0^i^	0^i^	0^i^	0^i^
F8	0^i^	0^i^	0^i^	0^i^
F9	0^i^	0^i^	0^i^	0^i^
F10	0^i^	0^i^	0^i^	0^i^
F11	0^i^	0^i^	0^i^	0^i^
F12	0^i^	0^i^	0^i^	0^i^

*Mean values (± SD) with different letters are significantly different (*p* ≤ 0.05; *n* = 3).

### HR-ESI MS and NMR spectroscopic analysis

**Figure 6:**
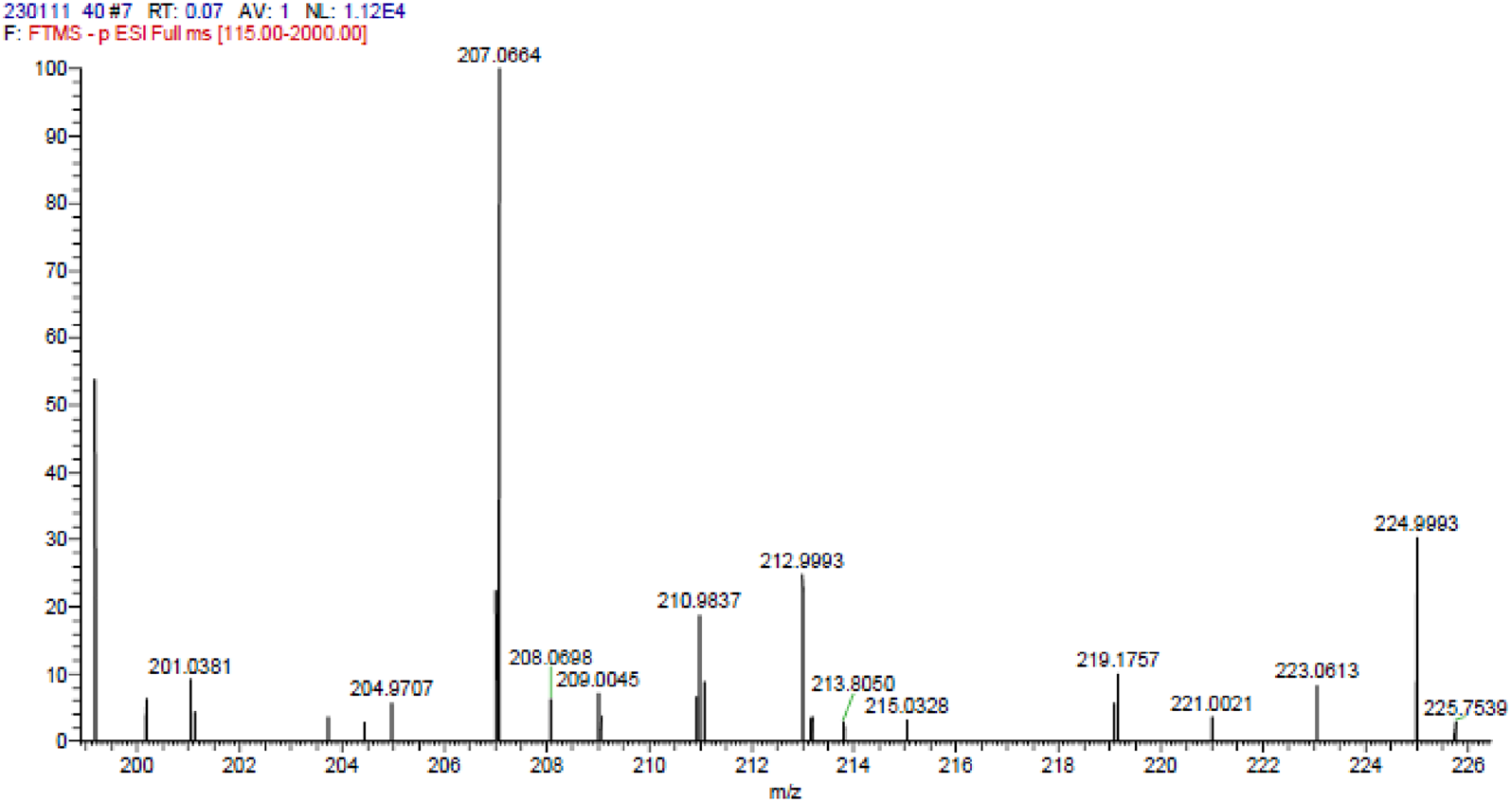
The HR-ESI–MS spectrum of compound 1.

**Figure 7:**
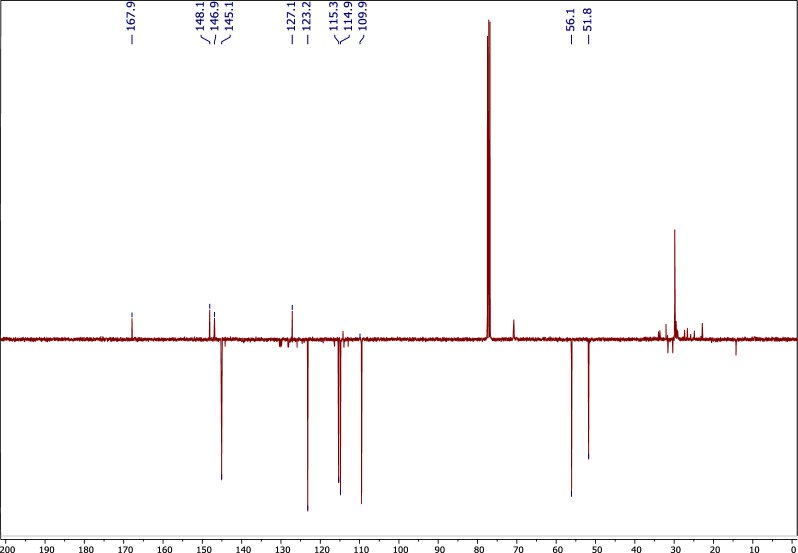
APT-NMR spectrum of compound **1** (100 MHz, CDCl_3_).

Compound **1** was isolated from *A. pseudodeflectus* AUMC 15761 as an off-white powder (8.0 mg), suggesting a molecular formula of C_11_H_12_O_4_ as deduced from its HR-ESI–MS spectrum (Fig. 6) which exhibited a [M−H]^−^ peak at *m/z* 207.0664 and a [M−H + H_2_O]^−^ peak at *m/z* 224.9995. The APT NMR spectrum of **1** (Table 3; Fig. 7), along with the HSQC analysis, confirmed the presence of 11 carbon atoms. These showed four quaternary carbons (including one carbonyl ester resonates at δ_C_ 167.8), five methines (including an olefinic double bond at δ_C_ 145.1 and 115.3), and two methoxy methyls (δ_C_ 51.7 and 56.0).

**Table 3:** ^1^H, ^13^C, and 2D NMR spectroscopic data of methyl ferulate **(1)** measured in CDCl_3_ (400 and 100 MHz, respectively).

No	*δ*_H,_ *(J* in H_z_)	*δ*_C,_ type	^1^H–^1^H COSY	HMBC
1	–	127.1, s	–	
2	7.02, 1H, d (2.0)	109.9, d	–	C-4, C-6, C-7
3	–	146.9, s	–	
4	–	148.1, s	–	
5	6.91, 1H, d (8.0)	114.8, d	H-6	C-1, C-3
6	7.06, 1H, dd (2.0, 8.0)	123.1, d	H-5	C-2, C-4
7	7.63, 1H, d (16.0)	145.1, d	H-8	C-2, C-6, C-8, C-9
8	7.28, 1H, d (16.0)	115.3, d	H-7	C-1, C-9
9	–	167.8, s	–	
10	3.79, 3H, s	51.7, q	–	C-9
11	3.92, 3H, s	56.0, q	–	C-3

**Figure 8:**
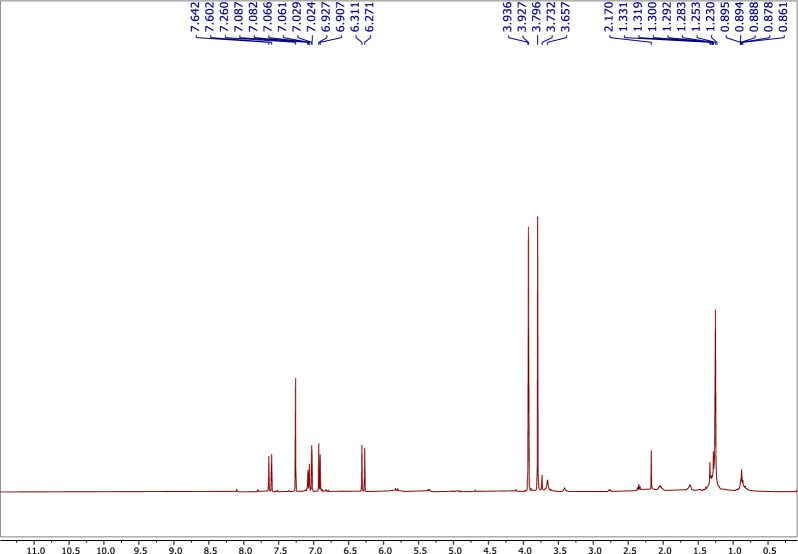
^1^H-NMR spectrum of compound **1** (400 MHz, CDCl_3_).

**Figure 9:**
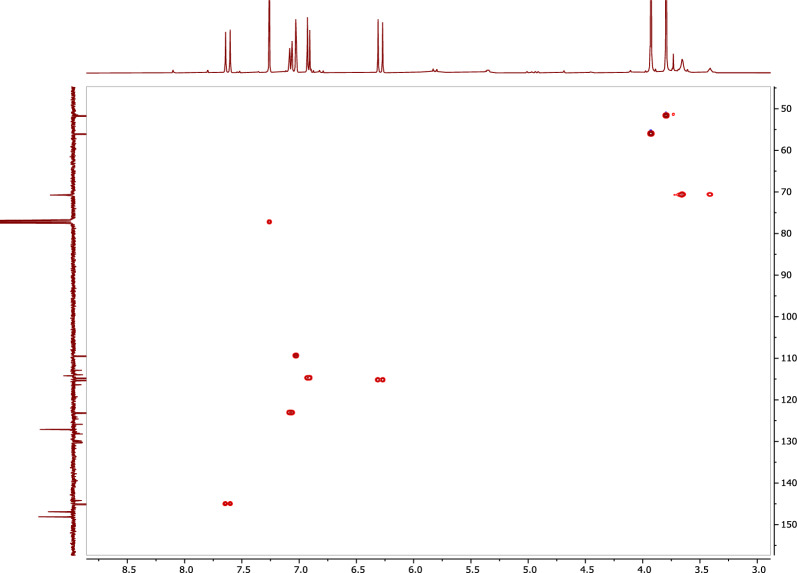
HSQC spectrum of compound **1** (400 MHz, CDCl_3_).

**Figure 10:**
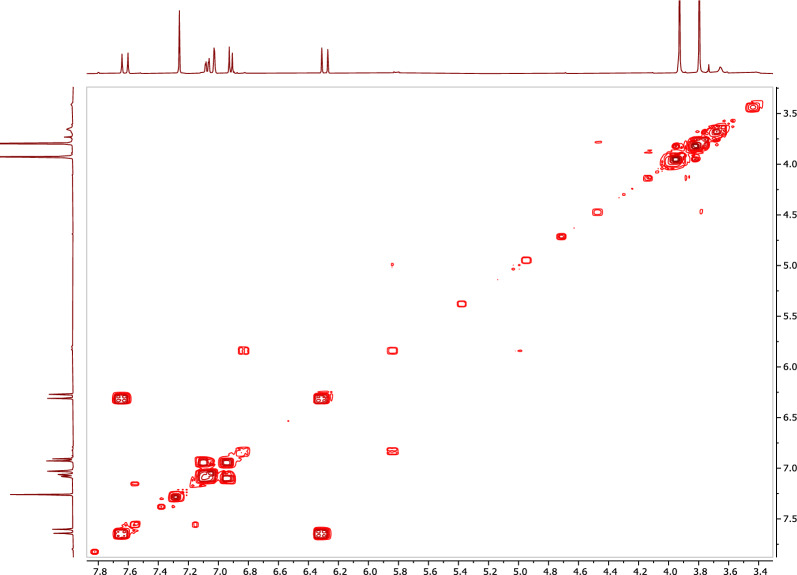
^1^H–^1^H COSY spectrum of compound **1** (400 MHz, CDCl_3_).

**Figure 11:**
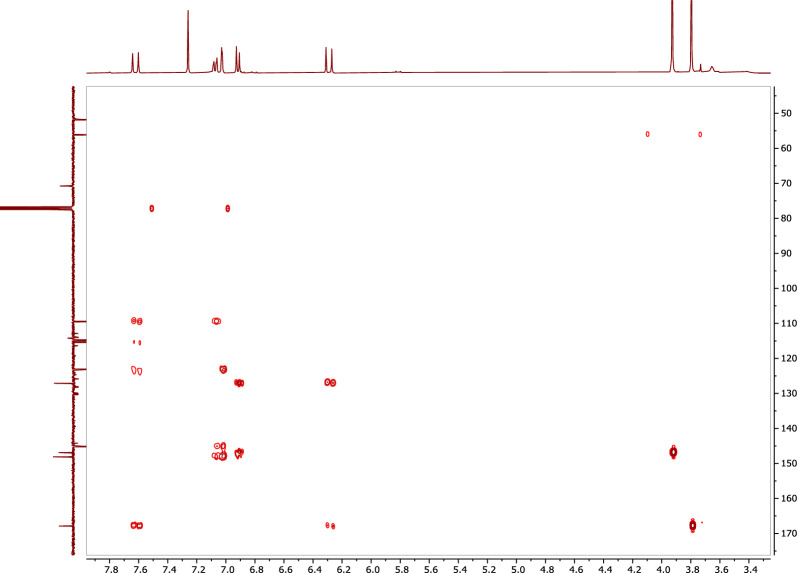
HMBC spectrum of compound **1** (400 MHz, CDCl_3_).

**Figure 12:**
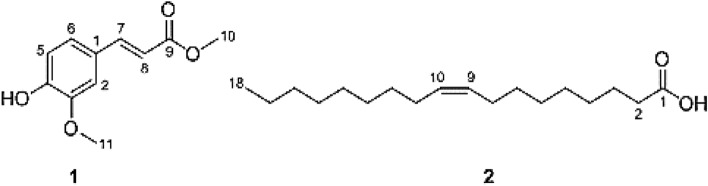
Chemical structure of methyl *trans*-ferulate (**1**) and oleic acid (**2**) produced by *A. pseudodeflectus* AUMC 15761 on wheat bran under SSF.

The ^1^H NMR spectrum of **1** (Table 3; Fig. 8), confirmed the presence of an olefinic double bond at δ_H_ 7.63 (1H, d, J = 16.0 Hz) and δ_H_ 7.28 (1H, d, J = 16.0 Hz). The HSQC correlation between the rest three methines and their corresponding protons [δ_C_ 109.9/δ_H_ 7.02 (1H, d, J = 2.0 Hz), δ_C_ 114.8/δ_H_ 6.91 (1H, d, J = 8.0 Hz), and δ_C_ 123.1/δ_H_ 7.06 (1H, dd, J = 2.0, 8.0 Hz)], along with the rest three quaternaries, revealed the presence of a tri-substituted benzene ring (Table 3; Fig. 9). The olefinic proton resonates at δ_H_ 7.28 and showed ^1^H-^1^H cosy correlations to the olefinic proton at δ_H_ 7.63 (Table 3; Fig. 10) and HMBC correlations with both the carbonyl carbon at δ_C_ 167.8 and the aromatic carbon at δ_C_ 127.1, indicating that **1** is a cinnamic acid derivative (Table 3; Fig. 11). The HMBC correlations between the methoxy protons [δ_H_ 3.79 (3H, s)] and the carbonyl carbon (δ_C_ 167.8) confirmed that **1** is a cinnamic acid methyl ester derivative. The other methoxy group at δ_H_ 3.92 (3H, s) showed a HMBC correlation with the quaternary aromatic carbon δ_C_ 146.9, and was downfield of the quaternary carbon δ_C_ 148.1, indicating that **1** is a ferulic acid methyl ester. The stereochemistry of the double bond was determined to be trans by the large coupling constant of its olefinic protons [δ_H_ 7.28 (1H, d, J = 16.0 Hz), at δ_H_ 7.63 (1H, d, J = 16.0 Hz)]. Based on this cumulative analysis, the structure of **1** was established as methyl ferulate (Fig. 12).

**Figure 13:**
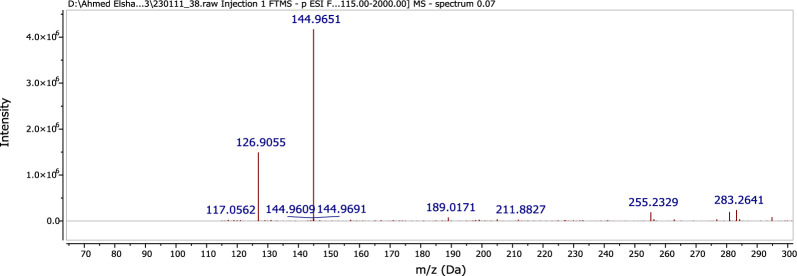
HR-ESI–MS spectrum of compound **2.**

**Figure 14:**
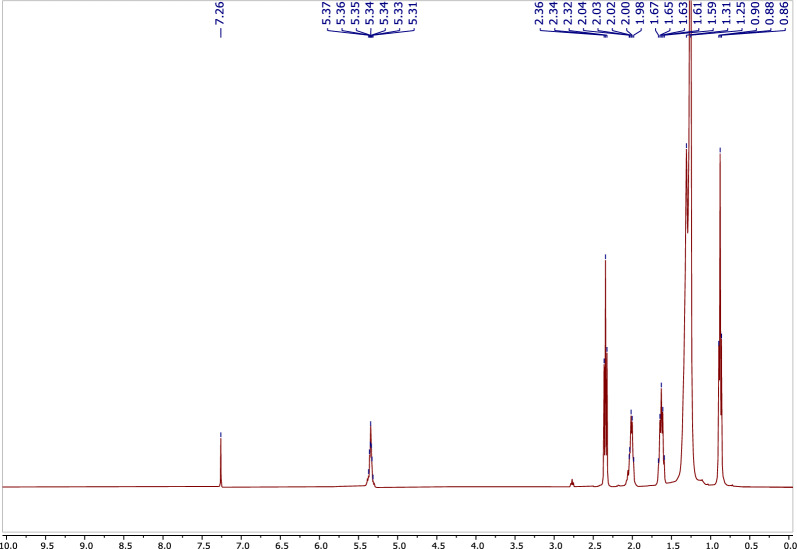
^1^H-NMR spectrum of compound **2** (400 MHz, CDCl_3_).

**Figure 15:**
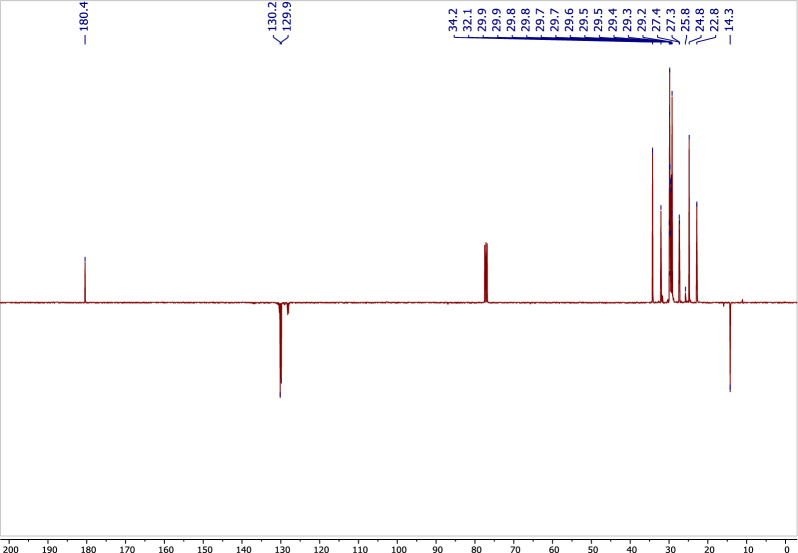
APT-NMR spectrum of compound **2** (100 MHz, CDCl_3_).

Compound **2** was isolated from *A. pseudodeflectus* AUMC 15761 as a colorless oil (40.0 mg). HR-ESI–MS (Fig. 13) suggested the molecular formula as C_18_H_34_O_2_ according to the mass peak [M + H]^+^ at *m/z* 283.2641. The ^1^H-NMR spectrum (Table 4; Fig. 14) exhibited a protons signal at δ_H_ 5.34 (2H, m, H-9, H–10) and δ_H_ 2.01 (4H, m, H–8, H–11), assignable to olefinic protons and allylic protons, respectively. The spectrum also revealed a methylene group α to carbonyl functionality (δ_H_ 2.34, 2H, t, J = 7.6, H-2). This was also substantiated by the APT NMR spectrum of **2** (Table 4; Fig. 15) which revealed the presence of a carbonyl carbon signal (δ_C_ 180.4, C–1), two olefinic carbon signals (δ_C_ 130.2 and 129.9, C–9, C–10), a group of methylene carbons resonances at δ_C_ 22.8–34.2, and finally a primary methyl group signal (δ_C_ 14.3, C–18), all of which were in agreement with a monounsaturated fatty acid. Thus, by comparing the ^1^H, ^13^C-NMR, and mass data of compound **2**, oleic acid was therefore determined to be the substance involved (Fig. 12).

**Table 4:** ^1^H, ^13^C-NMR spectroscopic data of compound **2**.

No	*δ*_H_, (*J* in Hz)	*δ*_C_, type
1		180.4, s
2	2.34, 2H, t, (7.6)	34.2, t
3	1.64, 2H, m	24.8, t
4–7, 12–17	1.25, 34H, m	22.8–32.1, t
8, 11	2.01, 4H, m	27.3, 27.4, t
9,10	5.34, 2H, m	129.9, 130.2, d
18	0.87, 3H, t, (7.2)	14.3, q

### Antioxidant activity of methyl ferulate and oleic acid produced by *Aspergillus pseudodeflectus* AUMC 15761

**Figure 16:**
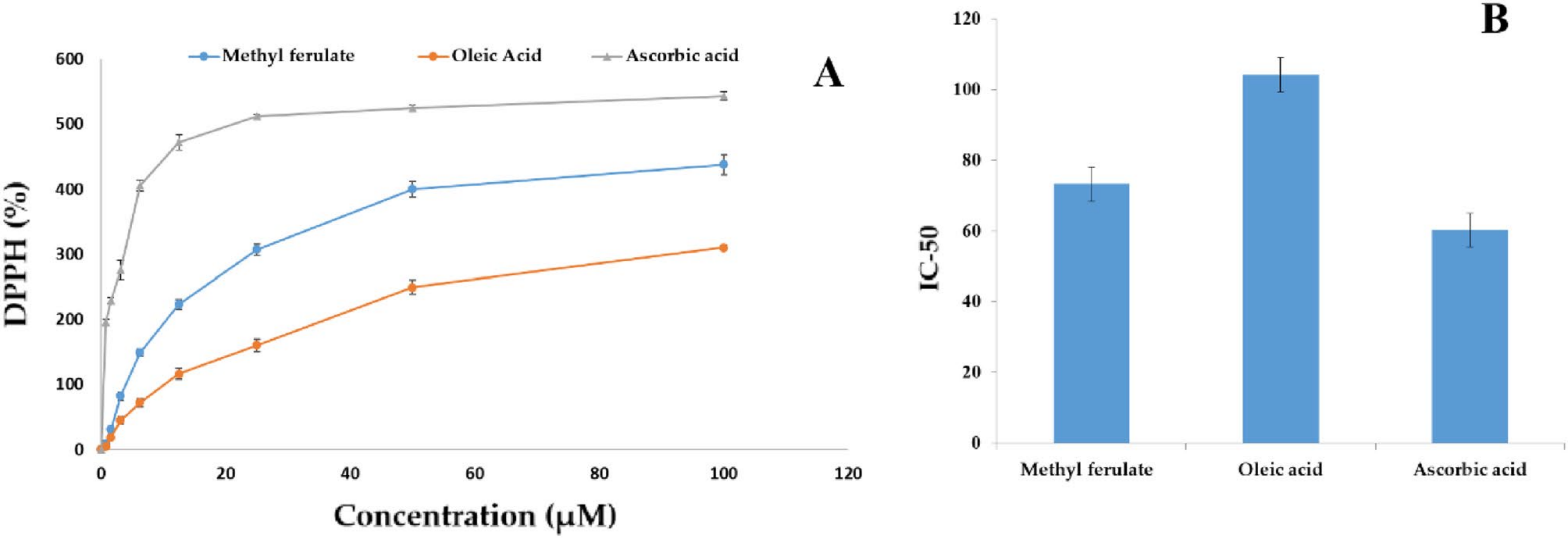
**(A)** Antioxidant activity (% DPPH) and **(B)** IC_50_ of methyl ferulate and oleic acid produced by *A. pseudodeflectus* AUMC 15761 compared to ascorbic acid as standard.

The results of the current study revealed two antioxidant substances—methyl ferulate and oleic acid. The DPPH activity increased as the sample concentration increased (Fig. 16A), with IC_50_ values of 73.213 ± 11.20 and 104.178 ± 9.53 µM were significantly greater (*p* < 0.05) respectively, for both substances, as compared to the value (60.299 ± 4.769 µM) of ascorbic acid (Fig. 16B; Table 5).

**Table 5:** The *t*-Test of pairwise comparison between methyl ferulate and oleic acid compared to ascorbic acid.

	Methyl ferulate	Oleic acid	Ascorbic acid
Mean	73.213	104.178	60.299
Variance	125.440	90.821	22.743
Observations	3.000	3.000	3.000
Pooled variance	108.130	56.782	
Hypothesized mean difference	0.000	0.000	
Df	4.000	4.000	
t Stat	-3.647	7.132	
P(T < = t) one-tail	0.011	0.001	
t Critical one-tail	2.132	2.132	
P(T < = t) two-tail	0.022	0.002	

### Antibacterial activity of methyl ferulate and oleic acid

**Figure 17:**
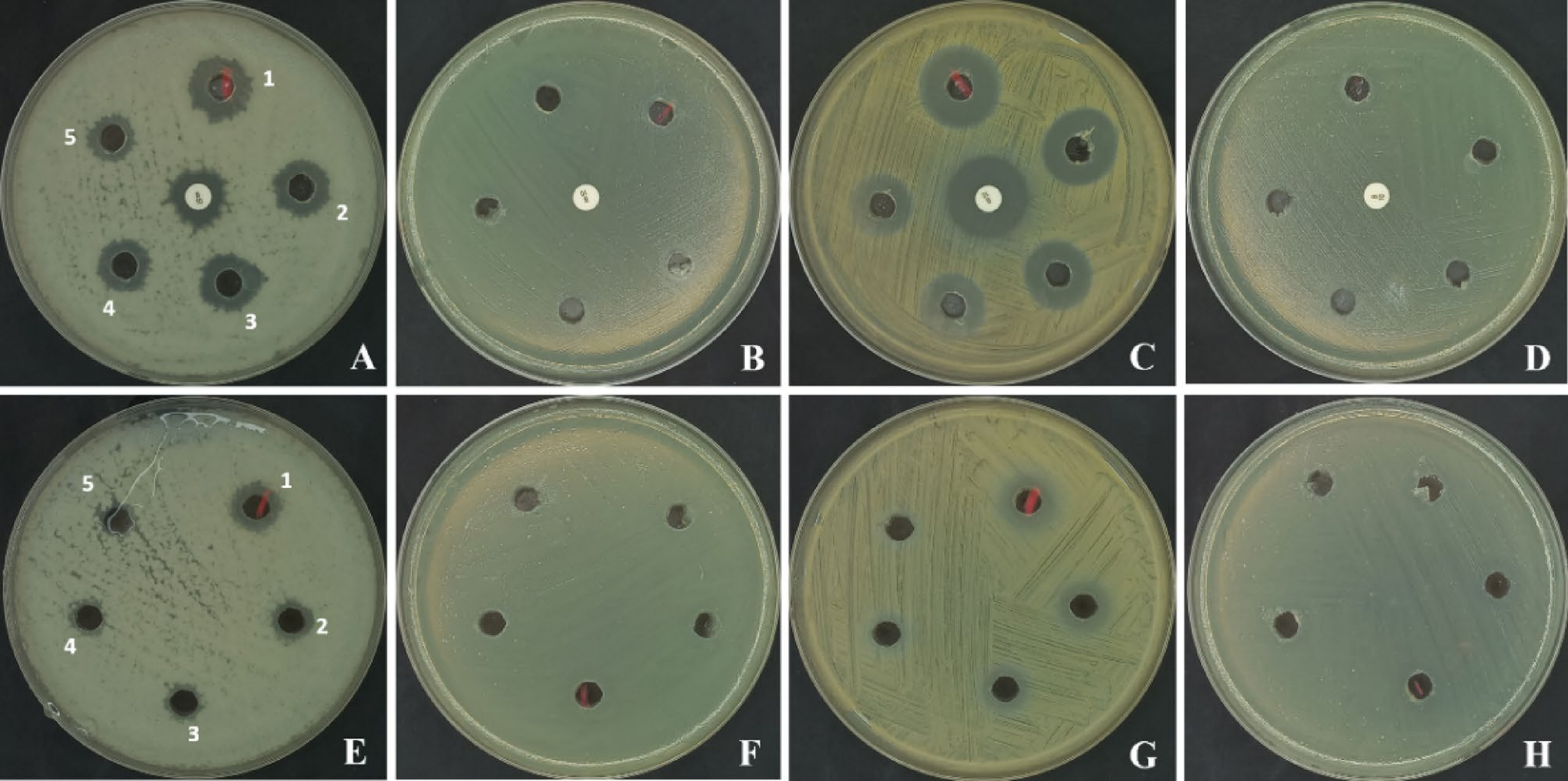
Antibacterial activity of the pure methyl ferulate and oleic acid produced by *A. pseudodeflectus* AUMC 15761 from wheat bran in SSF on **(A,E)**, *B. subtilis* **(B,F)**, *E. coli* **(C,G)**, *S. aureus* **(D,H)** and, *S. epidermidis*, respectively (1 = 5 µg/mL, 2 = 2.5 µg/mL, 3 = 1.25 µg/mL, 4 = 0.62 µg/mL, and 5 = 0.31 µg/mL).

In the present study, the antibacterial tests against *B. subtilis* and *S. aureus* demonstrated a significant antibacterial impact for both methyl ferulate and oleic acid. The MIC of methyl ferulate against the two bacterial strains was 0.31 mg/mL, while the MICs of pure oleic acid against both strains were 1.25 and 0.62 mg/mL, respectively (Tables 6, 7; Fig. 17).

**Table 6:** Antibacterial activity of pure methyl ferulate produced by *Aspergillus pseudodeflectus* AUMC 15761 from wheat bran in SSF compared to the Bacitracin antibacterial standard.

Tested bacteria	Purified Methyl ferulate (mg/mL)												Mean of Bacitracin (10 U) (MB)	*P value*	*R value*
	5		2.5		1.25		0.62		0.31		0.15				
	M	%MB	M	%MB	M	%MB	M	%MB	M	%MB	M	%MB			
*B. subtilis*	15.43	1.08	14.7	1.03	13.5	0.94	12.2	0.85	11.3	0.79	0	–	14.33	0.326	0.92
*E. coli*	0	–	0	–	0	–	0	–	0	–	0	–	0	–	–
*S. aureus*	20.27	0.97	18.23	0.87	16.5	0.79	15.23	0.73	14.6	0.69	0	–	21	0.337	0.98
*S. epidermidis*	0	–	0	–	0	–	0	–	0	–	0	–	0	–	–

For the data above, r equals 0.92, 0.98. This represents a very strong positive correlation. M = mean; %MB = mean value divided by the standard value.

**Table 7:** Antibacterial activity of pure oleic acid produced by *A. pseudodeflectus* AUMC 15761 from wheat bran in SSF compared to the Bacitracin antibacterial standard.

Tested bacteria	Purified oleic acid (mg/mL)										Mean of Bacitracin (10 U) (MB)	*P value*	*R value*
	5		2.5		1.25		0.62		0.31				
	M	%MB	M	%MB	M	%MB	M	%MB	M	%MB			
*B. subtilis*	11.5	0.8	9.67	0.67	8.33	0.58	0	–	0	–	14.33	0.41	0.833
*E. coli*	0	–	0	–	0	–	0	–	0	–	0	–	–
*S. aureus*	17.73	0.84	12.53	0.6	9.6	0.46	8.47	0.4	0	–	21	0.582	0.883
*S. epidermidis*	0	–	0	–	0	–	0	–	0	–	0	–	–

For the data provided above, r equals 0.833, 0.883. This represents a very strong positive correlation.

*Statistically significant at p < 0.05 (**) highly statistically significant at p < 0.01.

R = zero (no linear association between the variables or no consistent linear component to that relationship);R = 1 (perfect positive linear relationship between the variables); 0 < R < 1 (positive linear trend and the sampledindividuals are scattered around the line of best fit; the smaller the absolute value of R the less well the data canbe visualized by a single linear relationship. M = mean; %MB = mean value divided by the standard value.

*R* = zero (no linear association between the variables or no consistent linear component to that relationship); *R* = 1 (perfect positive linear relationship between the variables); 0 < *R* < 1 (positive linear trend and the sampled individuals are scattered around the line of best fit; the smaller the absolute value of *R* the less well the data can be visualized by a single linear relationship. M = mean; %MB = mean value divided by the standard value.

### Docking computations

**Figure 18:**
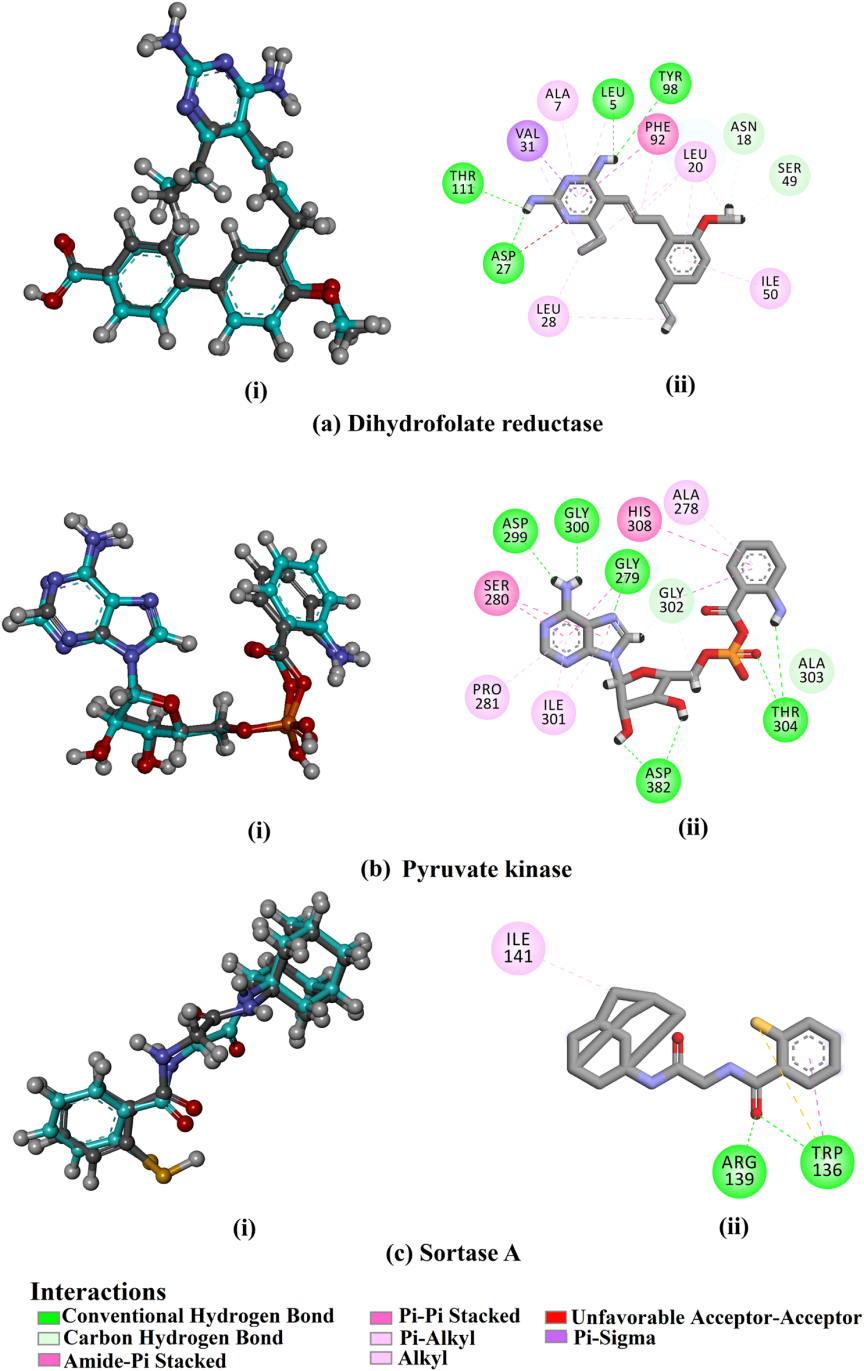
**(i)**, Superimposed structures of the experimental mode (in cyan) and the anticipated docking pose (in grey) **(ii),** 2D representation of the predicted binding modes of the co-crystalized ligands with the active site of different protein targets of *Staphylococcus aureus*.

In order to recognize target proteins for the antibacterial activity of the two compounds obtained in the present study, four various protein targets of *Staphylococcus aureus* were investigated, including dihydrofolate reductase (PDB code: 5ISP^32^), pyruvate kinase (PDB code: 5OE3^33^), and sortase A (PDB code: 2MLM^34^). Before generation of data, the performance of the AutoDock 4.2.6 software was evaluated via the re-docking of the co-crystalized inhibitors towards their targets. Based on the data illustrated in Fig. 18, the anticipated binding modes were essentially identical to their native structures with RMSD values less than 1.0 Å, displaying the strong accuracy of the utilized technique.

**Figure 19:**
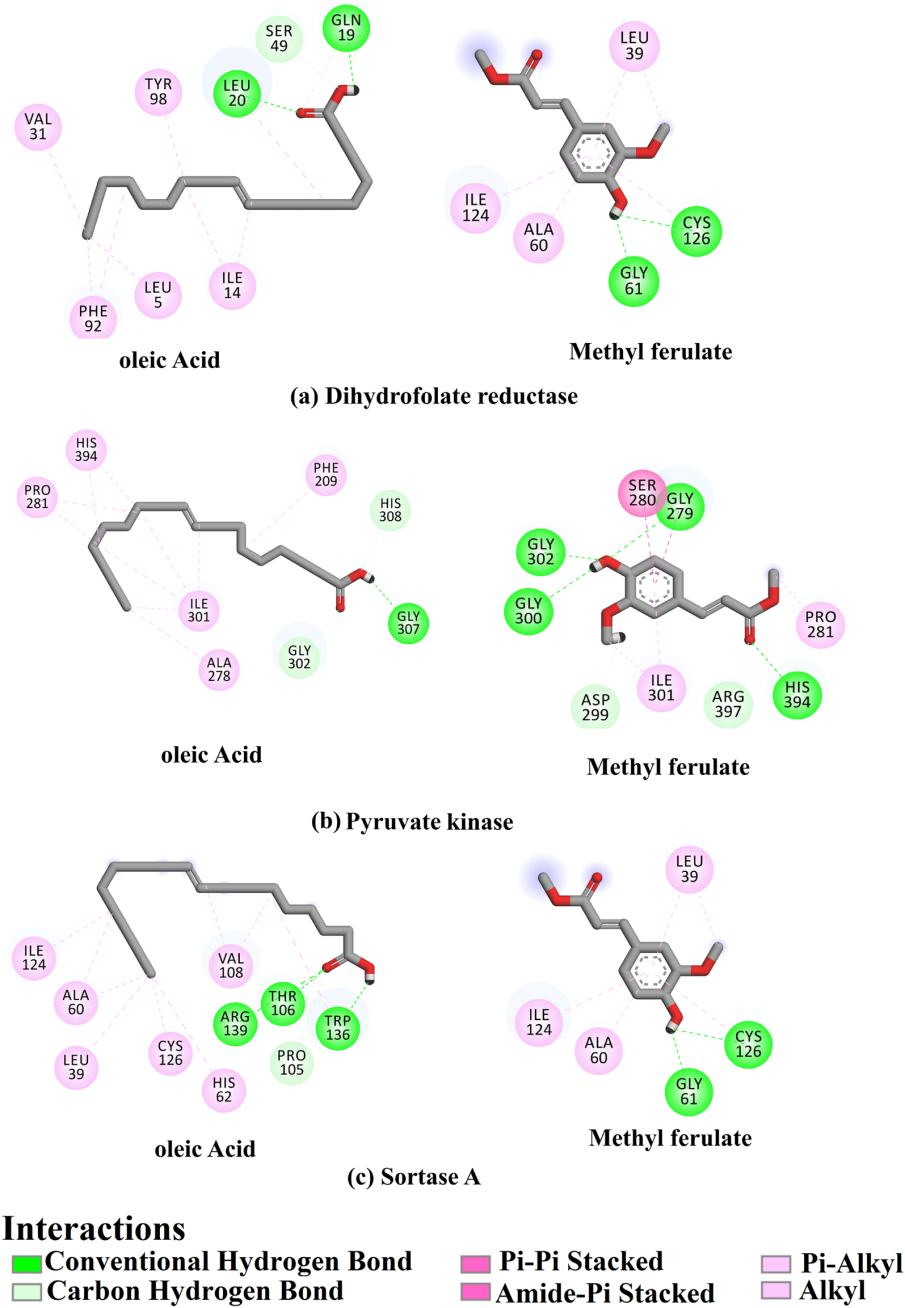
2D molecular interaction of the obtained compounds towards the active site of different protein targets of *Staphylococcus aureus*.

Using the assessed protocol, the docking pose of oleic acid and methyl ferulate with dihydrofolate reductase, pyruvate kinase, and sortase A was predicted and presented in Fig. 18. As illustrated in Fig. 19, oleic acid and methyl ferulate exhibited multiple H-bonds and additional interactions involving vdW (van der Waals), pi-based, and hydrophobic interactions. More precisely, oleic acid displayed an eminent docking score towards dihydrofolate reductase compared to methyl ferulate, with values of − 6.3 and − 5.7 kcal/mol, respectively (Table 8). Inspection the binding mode of oleic acid demonstrated that 2 H-bonds with GLN19 (1.78 Å) and LEU20 (2.18 Å). However, methyl ferulate exhibited 3 H-bonds with SER49 (1.68 Å), ALA7 (1.98 Å), and TYR98 (2.44 Å).

**Table 8:** Computed docking scores of the obtained compounds towards different protein targets of *Staphylococcus aureus*.

Compound	Docking score (kcal/mol)		
	Dihydrofolate reductase	Pyruvate kinase	Sortase A
Oleic acid	− 6.3	− 6.3	− 5.2
Methyl ferulate	− 5.7	− 5.7	− 4.9

For pyruvate kinase, oleic acid, and methyl ferulate, the exposed docking scores had values of − 6.3 and − 5.7 kcal/mol, respectively. Oleic acid formed a hydrogen bond with GLY307 (2.10 Å). In comparison, methyl ferulate exhibited four hydrogen bonds with HIS394 (2.26 Å), GLY302 (2.01 Å), GLY279 (1.88 Å), and GLY300 (2.16 Å). Sortase A, oleic acid and methyl ferulate displayed good docking scores with values of − 5.2 and − 4.9 kcal/mol, respectively. Oleic acid exhibited three hydrogen bonds with THR106 (1.99 Å), ARG139 (2.97 Å), and TRP136 (1.90 Å). On the other hand, methyl ferulate established two H-bonds with GLY61 (2.12 Å) and CYS126 (3.03 Å).

## Discussion

Secondary metabolites created by fungi have been shown to be a wonderful source of new drugs, biofuels, industrial chemicals, food additives, and feed additives^35^. Penicillins, lovastatin, echinocandin B, and cyclosporine A offer examples of how important it is to investigate fungal sources for novel pharmaceuticals^36^. Aromatic compounds, amino acids, fatty acids, butanolides, butenolides, cytochalasans, macrolides, naphthalenones, pyrones, and terpenes are only a few of the structural types of metabolites that fungi produce^36,37^. There have been 15600 fungal metabolites identified from species of *Alternaria*, *Aspergillus, Claviceps*, *Fusarium*, and *Penicillium*^38^.

In the present study, *A. pseudodeflectus* AUMC 15761 was identified based on sequencing of the ITS region. The fungus utilized wheat bran as a substrate in SSF to produce, for the first time, methyl ferulate and oleic acid, as confirmed by an antibacterial bioassay. Methyl ferulate was first reported from the plant *Stemona tuberosa*^14,15^ and then re-isolated from *Coriolopsis aspera*^39,40^, *Morinda citrifolia*^41^, and *Kigelia. africana*^42^. The *Aspergillus ustus* group has been found to produce several active metabolites such as the three novel isoquinoline alkaloids, TMC-120A-C (1–3) (furo [3, 2-h] isoquinoline-type) discovered from *A. ustus* TC 1118^43^; averufin, versicolorine C, along with austalides O and J have been isolated from different strains of *A. ustus*^44^; A newly isochroman derivative, pseudodeflectusin (9-hydroxy-7-methyl-2-(methylethylidine)-furano [3, 2-H] isochroman-3-one), was discovered from *Aspergillus pseudodeflectus*^45^; Drimane sesquiterpenoids 1, 15 were isolated from *Aspergillus pseudodeflectus* strains F-1860, F-1861^44^; Ophiobolin K and 6-epi-ophiobolin K, have been developed from *A. calidoustus* UFMGCB 4107^46^; Drimane sesquiterpenoids 1, 2, 7, and 15, TMC-120A-C, desferritriacetylfusigen and TMC-120 derivative 1, have been found in several strains of *A. calidoustus*^44^.

In addition, several bio-active secondary metabolites have been produced by many species of fungi in SmF or SSF^47^, such as penicillins and cephalosporins isolated from *Penicillium* and *Acremonium*, respectively^48,49^; feruloyl esterase produced by *Aspergillus terreus* GA2 using maize bran^50^; methyl ferulate from the fruiting bodies of *Coriolopsis aspera*^40^; festuclavine 2 produced by *A. fumigatus*^51^; cyclosporine 41, which exhibits broad spectrum of antifungal activity^52^, derived from *Toplypocladium inflatum*^53^; geranylgeraniol, farnesol, hexacosanol, oleic acid, and squalene synthesized by *Colletotrichum coccodes*^54^; 1-Octacosanol produced by *Phyllosticta capitalensis*^55^; a set of peptaibols, that are extremely potent growth inhibitors of several species of fungi, including the plant pathogens *Alternaria alternata*, *Phoma cucurbitaceum*, *Fusarium* spp., as well as the human pathogen *A. fumigatus*, have been reported from *Trichoderma reesei*^56^; and Echinocandin B 38 from *Aspergillus nidulans*^57^.

The most potent antibacterial crude metaboliate from *Aspergillus pseudodeflectus* AUMC 15761’s was produced after 10 days of fermentation utilizing wheat bran in SSF at pH 7.0 and 30 °C, using sodium nitrate as a nitrogen source. The pH of the growth medium and other physical factors, such as the incubation temperature, were found to have a substantial impact on the production of secondary metabolites, with synthesis rapidly decreasing on either side of an optimal point. By changing the degree of dissociation of different molecules in the media, the quantity of hydrogen or hydroxyl ions may have direct or indirect effects on a cell. As a result, variations in pH have an effect on the solubility and dissociation of intermediate products as well as the activity of microbial enzymes^58,59^.

One of the unsaturated fatty acids formed by the reaction of palmitic and stearic acids is oleic acid. Moreover, enzymatic activity can convert saturated fatty acids such as stearic acid and palmitic acid into oleic acid^60^. In the present study, the methanolic extract of wheat bran fermented by *A. pseudodeflectus* AUMC 15761 was utilized to isolate oleic acid. Saturated fatty acids and monounsaturated fatty acids, including palmitic and oleic acids, occurred in abundance in the fatty acid profiles of *Mucor circinelloides* URM 4140, *M. hiemalis* URM 4144, and *Penicillium citrinum* URM 4126^61^. Oleic acid, also known as 9-octadecenoic acid, is a healthy kind of omega-9 unsaturated fatty acid that is very useful for people's health^22^. Unsaturated fatty acids do indeed lower cholesterol by activating cholesterol acetyltransferase, as is widely known. Cancer, cardiovascular, autoimmune, Parkinson's, Alzheimer's, inflammatory, and hypertensive illnesses are all treated effectively with fatty acids. These compounds have been employed as an anticancer treatment because they may cause cancer cells to undergo apoptosis and regulate the cell membrane^22^.

*Aspergillus terreus*, *Claviceps purpurea*, *Tolyposporium* sp., *Mortierella alpina*, and *Mortierella isabellina* are a few examples of species of fungi that may collect lipids. Although several fungi may produce lipids, the majority of fungi are studied primarily for their capacity to create specific lipids such as docosahexaeneoic acid (DHA), gamma-linolenic acid (GLA), eicosapentaenoic acid (EPA), and arachidonic acid (ARA)^62^.

Bio-guided isolation of the secondary metabolites of the methanol extract of *A. pseudodeflectus* AUMC 15761 led to the purification of two active compounds—methyl ferulate and oleic acid. Their structures were determined by comparing their NMR and HR-EI-Ms data with that available from the literature^15,39,42^. It is noteworthy that this is the first report of producing oleic acid and methyl ferulate from *A. pseudodeflectus.*

Methyl ferulate and oleic acid in this study were found to have a significant antibacterial potential against gram positive bacteria, showing the best activity against *B. subtilis* and *S. aureus*, while *E. coli* and *S. epidermidis* were not affected by either compound in the present study. In the study, the MICs for methyl ferulate and oleic acid against *B. subtilis* and *S. aureus* were 79% and 69%, respectively, compared to the bacitracin standard, with MICs of 79% and 69%, and 58% and 40%, respectively. Similarly, the antibacterial activity of methyl ferulate against *Shigella putrefaciens* was determined^63^. Certain oils, including rose essential oil, have been proven to have antibacterial properties against *S. aureus*, and the efficacy against gram positive *S. aureus* was observed to be less than that of rifampin and gentamicin, with negligible MIC values^64^. Because of the observed sensitivity of gram-positive bacteria to the presence of one phenolic hydroxyl group in methyl ferulate, its antibacterial mechanism was taken into consideration^42,65^. Although the exact mechanism of the antibacterial activity of fatty acids is unknown, it is thought that their functional nature is connected to the permeability, membrane disruption, and fatal changes in the bacterial cytoplasm. As a result, rupture or alteration of the membrane-dependent conduction systems may occur^22,66^.* Escherichia coli*, a normally resistant bacterium, becomes very susceptible to the bactericidal effects of fatty acids if the lipopolysaccharide outer membrane is destroyed using ethylenediaminetetraacetic acid. As gram-negative bacteria, they are protected by their outer lipid membrane from the corrosive effects of oleic acid^67^.

Methyl ferulate has been reported to have antioxidant activity (% DPPH), with IC_50_ values of 73.213 11.20 µM, respectively^41^. Considering that, the principal mode of action of phenolic antioxidants is believed to be the scavenging of free radicals^68^. Due to the presence of one phenolic hydroxyl group in methyl ferulate, its antioxidant mechanism was taken into consideration^42,65^. Antioxidant activity (% DPPH) of OA with IC_50_ values of 104.178 ± 9.53 µM has been reported in the literature^69^. Methy ferulate is a new natural antibacterial agent with strong efficacy and low toxicity. It has great potential for applications in food preservation^63^. Oleic acid, which accounts for about 80% of the total fatty acids in virgin olive oil, has recently become an often used substance to protect food from oxidizing^24^.

Oleic acid and methyl ferulate both had positive docking results against several *Staphylococcus aureus* protein targets, based on the docking results. The development of hydrogen bonding interactions with the active site of the *Staphylococcus aureus* target proteins under investigation may be responsible for the high docking scores of these two compounds. These findings shed additional light on the significance of the chemicals that have been identified as potential candidates for antibacterial medication.

## Materials and methods

### Materials and chemicals

For the extraction, fractionation, and column chromatography, the organic solvents used were supplied by El-Nasr Pharmaceutical and Chemical Co. (ADWIC), Egypt. The deuterated chloroform (CDCl_3_) used for NMR analysis was purchased from Sigma-Aldrich. TLC pre-coated plates (F_254_ & PF_254_) and silica gel for column chromatography (70–230 and 230–400 Mesh) were provided by Merck (Darmstadt, Germany).

### Strain isolation and preservation

Using the dilution plate technique^70^, the strain for this investigation was isolated from a soil sample collected from Egypt's Aswan Governorate. Before adding Czapek's Dox agar (CzA) to Petri plates, the soil solution was appropriately diluted. The cultures were then maintained for two weeks at 25 °C. To create pure cultures of the fungus, the developed colonies were purified on CzA utilizing the single spore isolation technique^71^.

### Morphological and molecular identification of the *Aspergillus* strain

For morphological identification of the strain of *Aspergillus*, the fungus was inoculated on Malt Extract Agar (MEA), Czapek’s Yeast Autolysate Agar (CYA), and CzA^72^, and incubated for 7 days at 25 °C. The fungus in this study was morphologically identified on the basis of its macroscopic and microscopic characteristics, following the relevant key of *Aspergillus*: section Usti^73^. This strain was deposited and designated as AUMC 15761 in the culture collection of the Assiut University Mycological Centre. DNA isolation was carried out^74^, and the PCR reaction was performed by SolGent Co., Ltd (Daejeon, South Korea) using SolGent EF-Taq and the universal primers ITS1 and ITS4^75^. DNASTAR (version 5.05) was used to generate the contiguous sequences of the species of *Aspergillus* used in this investigation. There are 20 sequences in the overall ITS dataset that were used for phylogenetic analysis, consisting of one outgroup sequence for *Aspergillus creber* NRRL 58,592 (NR_135442), the sequence for *Aspergillus pseudodeflectus* (AUMC 15761 in this manuscript), and 18 sequences from the genus *Aspergillus*: section Usti acquired from GenBank. MAFFT (version 6.861b)^76^ with the default settings was used to align all sequences. Optimization of the alignment gaps and sparse uninformative characters was conducted by BMGE^77^*.* Maximum-likelihood (ML) and maximum-parsimony (MP) phylogenetic analyses were carried out using MEGA X (version 10.2.6)^78^, and 1000 replications^79^ were employed to assess the robustness of the most parsimonious trees. The ideal nucleotide substitution model for ML analysis was identified using Modeltest 3.7's Akaike Information Criterion (AIC)^80^. After editing, the tree was saved in TIF format^81^*.*

### Inoculum

*Aspergillus pseudodeflectus* AUMC 15761 was grown for 7 days on MEA at 25 °C and a spore solution (prepared in 10% tween 80) containing 1.5 × 10^8^ spores/mL was used to inoculate the substrate (5 mL/10 g substrate)^82^.

### Solid state fermentation (SSF) of lignocellulosic wastes

In order to determine how the selected isolate of *A. pseudodeflectus* produced an antibacterial chemical, five samples of agricultural waste were studied. The fermentation materials included barley bran (BB), date palm leaves (DPL), orange peels (OP), rice straw (RS), and wheat bran (WB). They were purchased from local marketplaces in Egypt's Assiut Governorate. Before being reduced to a size of 500 µm, they were cleaned with tap water to get rid of dirt and other impurities. As part of the pretreatment procedure, the samples were treated with 1.0% NaOH, thoroughly filtered, and then washed with tap water. They were then dried at 60 °C^83^. In order to determine how the selected *A. pseudodeflectus* produced an antibacterial chemical, five samples of agricultural waste were studied. Separate Erlenmeyer flasks (500 mL) were filled with 10 g of the lignocellulosic material, and the residue was then wetted down by 88% with a citrate buffer (pH 5.0). The flasks were next incubated for seven days at 30 °C.

### Extraction of bio-active compounds

Following the incubation time, the flask contents underwent 48 h of oven drying at 60 °C. The mycelial mat and solid substrate were stirred in 50 mL of methanol for 2 h at 180 rpm in each flask. The clear supernatant was obtained after centrifugation (10,000 rpm at 4 °C for 10 min). The volume of the methanol extract was then reduced by a rotary evaporator (Heidolph: Model reddot winner 2020; Germany). The sample was lyophilized into a powder using a freeze dryer (VirTis: Model 6 KBTES-55, NY; USA)^84^.

### Antibacterial effect of the crude extracts

The agricultural waste-derived crude extract residue from each sample was dissolved in dimethyl sulfoxide (DMSO) at a 10% concentration. The antibacterial efficacy of the crude extract was assessed using the agar well diffusion technique^85^, using 50 µL in each 5 mm well for *Escherichia coli* ATCC 8739, *Bacillus subtilis* ATCC 6633, *Staphylococcus aureus* ATCC 6538, and *Staphylococcus epidermidis* ATCC 12228. Bacitracin (10 U) and Piperacillin/Tazobactam 10: 1 (110 µg/disc), served as a positive control. This test was performed after each stage of purification.

### GC–MS analysis

This analysis was carried out at the Analytical Chemistry Unit (ACAL), Faculty of Science, Assiut University, Egypt. Before being injected into a GC–MS device (7890A-5975B; Thermo Scientific GC/MS; model ISQ; USA), with a nonpolar HP-5MS Capillary Standard column (30 × 0.25 × 0.25) mm, 0.5 g of the sample residue was dissolved in 5 mL of methanol and centrifuged for 15 min (10,000 rpm and 5 °C). The following was the cycle's parameters: oven program on at 120 °C for 5 min, 30 °C/min rising to 265 °C for 25 min, then 50 °C/min increased to 280 °C for 5 min; run duration 48 min; post run 260 °C for 2 min; flow program 0.5 mL/min. for 10.9 min., and then 1 mL/min for 30 min. Equilibration time was 0.5 min, and the maximum temperature 280°C^84^.

### Optimization of bio-active secondary metabolites production by *A. pseudodeflectus* AUMC 15761

For maximization of secondary metabolites production, the respective pH (4.0, 5.0, 6.0, 7.0, and 8.0), temperature (20, 25, 30, 35, and 40 °C), nitrogen source (peptone, yeast extract, sodium nitrate, ammonium chloride, and ammonium sulphate, each at 0.2%), incubation time (2, 4, 6, 8, up to 14 days) were adjusted using one factor at a time (OFAT)^84^. For the testing, a 10 g amount of wheat bran was placed in 500 mL Erlenmeyer flasks. Following the incubation time, the bio-active Secondary metabolites were extracted as described above and then used in further tests.

### Production of bio-active secondary metabolites by *A. pseudodeflectus* AUMC 15761 utilizing wheat bran in SSF

Ten g portions of wheat bran were separately placed into 500 mL Erlenmeyer conical flasks, and the spore suspension (1.5 × 10^8^ spore/mL) was prepared from a seven-day-old *A. pseudodeflectus* AUMC 15761 culture and added to each flask in a volume of 5.0 mL were used to inoculate the residue. The flasks were then kept under optimal production cultural conditions. Following the incubation time, the flask contents were extracted, and the dried extract was applied throughout the purification process.

### Purification of the secondary metabolites by column chromatography

#### Sample preparation for column chromatography

The residue was combined with an equivalent weight of silica gel powder and a trace amount of methanol was added to create a slurry prior to each step of purification. The extract was placed onto a vacuum liquid chromatography (VLC) column for fractionation after being dried and slurred. The last stage of purification was carried out by adding the obtained fractions to silica gel column chromatography^84^.

#### Vacuum liquid chromatography (VLC)

With 900 g of silica gel (230–400 Mesh), the entire crude extract was fractionated using a VLC column (5.0 × 120 cm). The *n*-hexane, Dichloromethane (DCM) and 0–100% gradients of DCM in MeOH (by adding 10.0% MeOH each time) were used in the fractionation process. A low-pressure evaporator was used to dry out the solvents after collecting a total of 250 mL of elutes. Fractions that produced the strongest antibacterial properties and have comparable spots were combined, condensed, and dried for use in the further purification process.

#### Thin layer chromatograph (TLC)

TLC was carried out on pre-coated silica gel F_254_ plates. A series of solvents of increasing polarities were used for developing the spots. For visualization of the spots, the plates were subjected to a UV inspection (at 365 and 254 nm) and then sprayed with 10% (v/v) H_2_SO_4_ in methanol, dried using a hot air drier, and heated to 110°C^84^.

#### Final purification of the active secondary metabolites

The fraction containing active chemicals was subsequently chromatographed on an open column (1.0 × 100 cm) equipped with a 35 g silica gel (70–230 Mesh). It was eluted in *n*–hexane gradients with 0–20% EtOAc (adding 1.0% EtOAc each time). TLC was used to detect 25 mL elutes using three mobile systems (*n*–hexane: EtOAc: 95: 5, 90: 10, and 80: 20). After combining and drying similar elutes, nine subfractions were obtained. The subfractions containing the most active compounds were uploaded over a second column (0.5 × 25 cm) packed with 15 g silica gel and a solvent system of n–hexane: acetone (95: 5) was used for elution. A 15 mL elutes were collected, subjected to TLC, and those containing nearly pure compounds were combined. Following the method of Siddiqui, et al.^86^, they were finally purified using preparative TLC plates (60 PF_254_). The antibacterial test for the purified compound was performed as described above.

### Spectroscopic NMR

The analysis was completed at the Micro-Analytical Unit (MAU) of Cairo University's School of Pharmacy in Giza, Egypt. Bruker Avance III HD 400 and 100 MHz spectrometers (Bruker Biospin, Rheinstetten, Germany) and NMR software Topspin 3.2 pl 6 were used to produce the ^1^H and ^13^C-NMR spectra. The internal reference standard was tetramethylsilane (TMS). An LTQ Orbitrap XL spectrometer was used to obtain the HR-ESI–MS data (Thermo Fisher Scientific; USA).

### DPPH radical scavenging activity

Using the methods described by Yen and Duh^87^, a freshly prepared methanol solution containing 0.004% (w/v) of the 2,2-diphenyl-1-picrylhydrazyl (DPPH) radical was refrigerated at 10 °C in the dark. Pure compound concentrations of 100, 50, 25, 12.5, 6.25, 3.125, 1.56, and 0.78 µM methanol were utilized for the pure synthesized sample, respectively. The absorbance at 515 nm was estimated using a 40 µL fraction of the sample-containing methanol solution combined with 3 mL of the DPPH solution (Milton Roy, Spectronic 1201; Canada). The decrease in the absorbance at 515 nm was monitored continuously until it stabilized, with data obtained at 1 min intervals for 16 min. The absorbance of the DPPH radical without an antioxidant was measured, as was the absorbance of ascorbic acid, a reference chemical. The percentage inhibition (PI) of the DPPH radical was estimated using the Eq. (1):

1 $${\text{PI}} = \left( {{\text{AC}} - {\text{AT}}} \right) \times {1}00$$

where AC is the control absorbance at time zero and AT is the sample absorbance plus DPPH at 16 min. The dose–response curve graphic plots were used to estimate the 50% inhibitory concentration (IC_50_), which is the concentration necessary to inhibit the DPPH radical by 50%. The experiment was carried out in triplicate.

### Computational methods

The crystal structures of dihydrofolate reductase (PDB code: 5ISP^32^), pyruvate kinase (PDB code: 5OE3^33^), and sortase A (PDB code: 2MLM^34^) were obtained and utilized as templates for all *in-silico* computations. To prepare these proteins, all inhibitors, ions, and water molecules were removed. Modeller software was employed to construct all missing residues^88^. In addition, the H +  + website was utilized to inspect the protonation states of the studied targets^89^. All missing hydrogens were added. The structure of the investigated inhibitors was manually built. Prior to docking computations, all inhibitors were minimized using the MMFF94S force field within SZYBKI software^90,91^. The atomic charges of the obtained compounds were determined utilizing the Gasteiger method^92^. All docking calculations were conducted using AutoDock4.2.6 software^93^. Based on the AutoDock protocol, the investigated targets were saved in pdpqt format^94^. The number of genetic algorithm (GA) runs was adjusted to 250. Moreover, energy evaluations (*eval*) were set to 25,000,000. The default settings of the other docking parameters were utilized. The grid box was set out to include the active site for all investigated targets.

### Statistical analysis

The experimental data were recorded as an average value and SD, and each test was done in three replicates. The Duncan's multiple range test was performed after the analysis of variance (ANOVA: two-factor with replication) and (t-Test: Two-Sample Assuming Equal Variances) on the data^95,96^.

## Conclusions

In this study, *Aspergillus pseudodeflectus* AUMC 15761 used wheat bran in SSF to produce methyl ferulate and oleic acid for the first time, both of which have strong antibacterial and antioxidant properties. The most prevalent chemical components were determined using gas chromatography-mass spectrometry (GC–MS) analysis of the crude methanol extract. An in-depth spectroscopic investigation was used to identify methyl ferulate and oleic acid, which were then purified using column chromatography. Significant antioxidant and antibacterial properties of the two compounds against *Bacillus subtilis* and *Staphylococcus aureus*, respectively, were detected. These findings were ditto analyzed *in-silico* utilizing the molecular docking technique. According to docking computations, methyl ferulate, and oleic acid demonstrated good antibacterial activity.

## Data Availability

All data related to this manuscript are incorporated in the manuscript only.
