# Author Correction: Antioxidant, antibacterial, and molecular docking of methyl ferulate and oleic acid produced by *Aspergillus pseudodeflectus* AUMC 15761 utilizing wheat bran

**DOI:** 10.1038/s41598-024-58336-9

**Published:** 2024-04-08

**Authors:** Ahmed Mohamed Ahmed Ali Ramadan, Sabry Ahmed Hussein Zidan, Reda Mohamed Shehata, Hussein Hosny EL‑Sheikh, Fuad Ameen, Steven L. Stephenson, Osama Abdel‑Hafeez Mohamed Al‑Bedak

**Affiliations:** 1 Department of Botany and Microbiology, Faculty of Science, Al Azhar University, Cairo, Egypt; 2 Department of Pharmacognosy, Faculty of Pharmacy, Al-Azhar University, Assiut Branch, Assiut, 71524 Egypt; 3 The Regional Center for Mycology and Biotechnology (RCMB), Al Azhar University, Cairo, Egypt; 4 Department of Botany and Microbiology, College of Science, King Saud University, 11451 Riyadh, Saudi Arabia; 5 Department of Biological Sciences, University of Arkansas, Fayetteville, USA; 6 Assiut University Mycological Centre, Assiut University, Assiut, 71511 Egypt

Correction to: *Scientific Reports* 10.1038/s41598-024-52045-z, published online 07 February 2024

The Funding section in the original version of this Article was incomplete. It now reads:

"Open access funding is provided by The Science, Technology & Innovation Funding Authority (STDF) in cooperation with The Egyptian Knowledge Bank (EKB). This research was also funded by Researchers Supporting Project number (RSP2024R364), King Saud University, Riyadh, Saudi Arabia.”

The original Article has been corrected.

